# Bidirectional associations between parental negativity and child externalising problems: Social support and neighbourhood cohesion as moderators

**DOI:** 10.1002/jcv2.70054

**Published:** 2025-10-08

**Authors:** Jasmine A. L. Raw, Jon Heron, Bonamy R. Oliver, Jane Gilmour, Emily Midouhas

**Affiliations:** ^1^ Psychology and Human Development Institute of Education University College London London UK; ^2^ Population Health Sciences Bristol Medical School University of Bristol Bristol UK; ^3^ Great Ormond Street Institute of Child Health University College London University College London UK

**Keywords:** ALSPAC, behaviour problems, parent–child relationships, random intercept cross‐lagged panel model, social support

## Abstract

**Background:**

Externalising behaviours are among the most common childhood mental health problems and have been linked to numerous adverse psychosocial outcomes including antisocial behaviour and depression. Parental negativity (PNeg) and child behaviours have been shown to mutually influence each other, leading to coercive cycles of negative behaviour over time. Interrupting these negative cycles is a common target for clinical intervention but little is known about what factors moderate these cycles over time in the general population.

**Method:**

Using data on 9943 families from The Avon Longitudinal Study of Parents and Children across ages 4, 7 and 8, we explored the reciprocal associations between PNeg and externalising behaviour and tested whether they differed as a function of high versus low parent‐reported interpersonal social support and neighbourhood social cohesion.

**Results:**

Using random‐intercept cross‐lagged panel models, we found bidirectional associations between PNeg and child externalising behaviour across ages 7 to 8 (*β*s = 0.13–0.15) but not ages 4 to 7 (*β*s = 0.01–0.03). Moreover, we did not find evidence of moderation of any of the cross‐lagged paths by social support or neighbourhood cohesion.

**Conclusions:**

Parent‐reported interpersonal social support and neighbourhood social cohesion do not appear to play a role in interrupting negative parent–child interaction cycles in the general population.

## Key points

**What's known?**

- The bidirectional relationship between parental negativity (PNeg) and child behaviours can sometimes lead to coercive cycles of negative behaviour. While clinical interventions often target these negative cycles, little is known about what factors might disrupt them in the general population.

**What's new?**

- We tested the bidirectional associations between PNeg and child externalising problems when children were aged 4, 7 and 8. We also looked to see whether this relationship was moderated by interpersonal social support and neighbourhood social cohesion.
- Bidirectional associations between PNeg and child externalising behaviour were found across ages 7 to 8 but not ages 4 to 7.

**What's relevant?**

- Contrary to expectations, social support and neighbourhood cohesion did not appear to play a role in interrupting parent–child interaction cycles.

## Graphical abstract

This study, using data from Avon Longitudinal Study of Parents and Children across ages 4, 7 and 8, found bidirectional associations between parental negativity and child externalising behaviour across ages 7 to 8 but not ages 4 to 7. Contrary to expectations, social support and neighbourhood cohesion did not moderate any of the cross‐lagged paths.

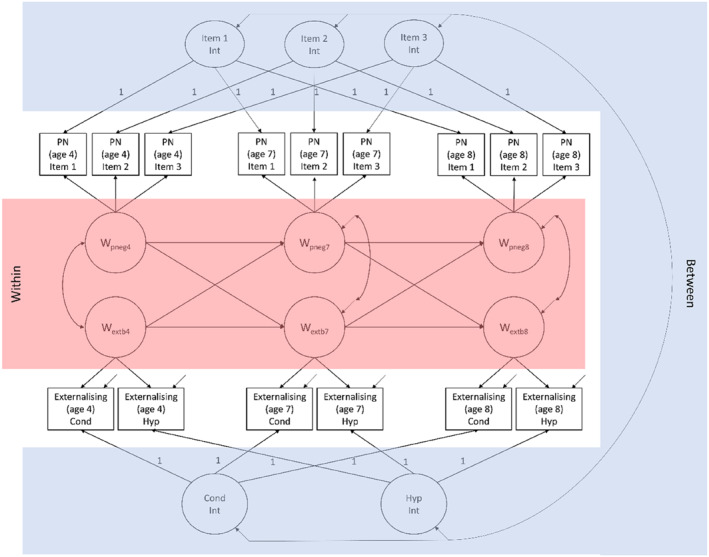

## INTRODUCTION

Externalising behaviours (e.g., conduct problems, aggression) are one of the most common mental‐health problems in childhood (Sacco et al., 2022), predicting adverse outcomes, including poor academic achievement, substance use, lack of employment and criminality (Colman et al., 2009; Erskine et al., 2016). Associations between externalising behaviour and parent–child relationships are well‐established. For example, PNeg (controlling or hostile feelings and behaviours) is associated with short‐ and long‐term child externalising outcomes (Pinquart, 2021; Rothenberg et al., 2020; Wiggins et al., 2015) and children's externalising behaviour predicts subsequent PNeg (Yan et al., 2021). Bidirectional parent‐ and child‐driven effects are present across multiple studies (Flouri et al., 2016; Speyer, Hang, et al., 2022) and are robust to genetic confounds (Oliver, 2015). This bidirectionality is of great interest in the intervention context for children's behavioural problems, since coercive parent–child interaction patterns are theorised to be primary mechanisms for the onset and maintenance of these problems. Specifically, coercion theory (Patterson, 1992) describes a process of mutual reinforcement where parents inadvertently bolster their children's challenging behaviours, leading to parents displaying negativity, and a subsequent perpetuating parent–child cycle until either the parent or the child gives in. This pattern can be embedded within families over time, elevating the risk of conduct problems in children. Parent‐training programs that aim to break negative––and increase positive––patterns between parents and children are recommended best practice (Shelleby & Shaw, 2014).

We know little about social contextual factors that may interrupt parent–child negative behaviour cycles in the general population, that is, outside of the intervention context. The diverse response of parents and children to contextual risks (Kim‐Cohen et al., 2004) suggests that such risks may play a part in moderating patterns of behaviour between parents and children. Research has identified vulnerability factors important for exacerbating coercive parent–child cycles in the general population, such as maternal depression, child temperament and genetic risk of psychopathology (Trentacosta et al., 2019). However, to our knowledge, no community‐sample studies have attempted to identify factors in families' social environments that specifically help parents and children to engage in fewer disproportionately negative interactions, thereby reducing coercive cycles. Here, we examine the role of two distinct aspects of the social environment as potential moderators of parent–child bidirectional processes and consider these factors as potential buffers for children from the deleterious effects of PNeg and for parents from the deleterious effects of children's externalising behaviour.

The social environment plays a central role in the parenting process (Belsky, 1984; Taraban & Shaw, 2018), a key aspect of which is interpersonal social support, a multidimensional concept defined as a psychosocial resource available in the context of an individual's social network (Moak & Agrawal, 2009). Three main forms of social support are considered in the literature: emotional (e.g., encouragement, nurturance), informational (e.g., advice, information) and instrumental (e.g., financial assistance) (Taylor, 2011). For parents, a variety of sources may provide social support, including romantic partners, extended family, and close friends and neighbours, with higher levels of support linked to increased parental warmth and sensitivity (Lee et al., 2020; Lippold et al., 2018) and decreased parental hostility and over‐reactivity towards the children (Lippold et al., 2018).

Interpersonal social support could buffer the impact of negative parenting on children's behaviour (Conger & Conger, 2002; Taraban et al., 2019) by reducing parental stress and allowing parents to parent more positively (Liu et al., 2020). Since prior research indicates that social support is associated with parental warmth (Lippold et al., 2018), when considering the moderation of parent‐to‐child effects, we expect a concurrent and longer‐term buffering of the effect of negative parenting on children's behaviour in the context of greater social support. For example, greater social support could mean that there are more adults in the family's social network to help with the child, thereby minimising children's exposure to negative parenting, but also, importantly, offering respite for parents at risk of negative parenting, which may limit their negativity towards their children (Horton, 2003). Supporting this notion, emotional social support has been shown to disrupt the continuity of parental maltreatment (Conger et al., 2013), and reduce parental stress (Armstrong et al., 2005) which may increase positive and decrease negative parenting (Lippold et al., 2018), in turn lowering children's behavioural problems (Akcinar & Baydar, 2016). Turning to the moderation of child‐to‐parent effects, prior findings suggest that interpersonal social support in the form of perceived high‐quality coparenting can moderate the negative effects of children's externalising behaviour on parent's sense of competence (Latham et al., 2017), with likely knock‐on effects for the coercive cycle. Moreover, greater social support from friends and partners may mean more opportunities for children to have a positive relationship with a different adult who may, for example, offer them encouragement and praise (Heberle et al., 2015) and which, in turn, may reduce their behavioural problems. We expect social support to have moderating effects on the interplay between PNeg and children's behaviour concurrently and across time, reducing parent over‐reactivity with knock‐on effects to children's behaviour, as well as being a protective factor for parents in the face of their children's challenging behaviour.

A more distal aspect of the social environment that could also moderate the bidirectional parent–child process is neighbourhood social cohesion (Cuellar et al., 2015; Tendulkar et al., 2010). Neighbourhood social cohesion is defined as the set of shared norms, trust, and networks within a community, reflecting the quality and quantity of social interactions between neighbours (Forrest & Kearns, 2001). Where interpersonal social support and neighbourhood cohesion might slightly overlap is in relation to perceptions of neighbours as a source of support and social interaction if neighbours are considered to be part of one's interpersonal social network. Perceptions of neighbourhood social cohesion have been associated with individual mental‐health and wellbeing outcomes (Breedvelt et al., 2022; Kim et al., 2024), evidencing a dose‐response effect on mental health (Solmi et al., 2017), as well as parenting (Cuellar et al., 2015; Tendulkar et al., 2010). Moreover, neighbourhood cohesion may buffer the link between PNeg and children's externalising behaviour (Silk et al., 2004). Yet, neighbourhood social cohesion is rarely explored as a possible buffer of reciprocal parent–child processes. We expect that the wider social network afforded to parents and children in more socially cohesive neighbourhoods (Breedvelt et al., 2022) may offer increased opportunities for monitoring and looking after children as well as reducing parental stress (Chung & Steinberg, 2006), with subsequent reductions in children's externalising behaviours.

In the present study, we explored bidirectional associations between PNeg and child externalising behaviours across ages 4, 7 and 8 using data from a UK cohort study, the Avon Longitudinal Study of Parents and Children (ALSPAC). We modelled bidirectional associations within families using Random‐Intercept Cross‐Lagged Panel Models (RI‐CLPM) to reflect the interplay between parents and children that characterise Patterson's coercion model whereby parents and children mutually reinforce negative (parent) or challenging behaviours (child) (Patterson, 1992; Speyer, Hang, et al., 2022). Additionally, we examined whether parental interpersonal social support and neighbourhood social cohesion moderated these parent–child processes. We expected to find bidirectional associations between PNeg and child externalising behaviour, and differences in the parent‐driven and child‐driven effects within families for those exposed to more, compared with less, interpersonal social support and neighbourhood social cohesion.

## METHODS

### Participants and procedure

ALSPAC is an ongoing transgenerational longitudinal cohort study that originally invited pregnant women residing in Avon, UK who were due to give birth between 1 April 1991 and 31 December 1992 (http://www.bristol.ac.uk/alspac/researchers/our‐data/). ALSPAC allows for the investigation of social, biological, and environmental impacts on pregnancy outcomes and child mental and physical health (Boyd et al., 2012; Fraser et al., 2013). Initially, 14,541 pregnancies were enroled into the study (including 14,203 unique mothers and 14,062 live births) of which 13,988 children were alive at 1 year of age. For our study, we used data obtained from mothers who were enroled into the original (Core) ALSPAC cohort who had singleton children that were alive at the age of 1 and had not withdrawn consent (*N* = 13,564; see Figure S1). We measured PNeg and child externalising behaviours using postal questionnaires administered to the study child's main carer at around ages 4 years (47 months), 7 years (81 months) and 8 years (97 months). Our moderators, interpersonal social support and neighbourhood social cohesion, were assessed at 5 years (61 months) and were temporally closest to the baseline variables measured at age 4 (47 months). Covariates include child sex assigned at birth and indicators of family adversity. Further details of the data available in ALSPAC can be found via the data dictionary: http://www.bristol.ac.uk/alspac/researchers/our‐data/.

We included families (*n* = 9943) with data on both PNeg and externalising behaviour in at least one of three timepoints (child ages 4, 7 and 8). In our sample, 94% were of White ethnic background and 49% of the children were female (sex assigned at birth). In addition, 12% of the sample reported low parental education and the percentage of families experiencing at least one adversity ranged from 5.7% (early parenthood) to 35.8% (maternal psychopathology; See Table S2 for additional sample demographics). We also report our results with the complete case sample found in Supporting Information S1 (*n* = 5394).

### Ethical considerations

Ethical approval for the study was obtained from the ALSPAC Law and Ethics Committee (IRB00003312) and local Research Ethics Committees (see https://www.bristol.ac.uk/media‐library/sites/alspac/documents/governance/Research%20Ethics%20Committee%20approval%20references.pdf). Informed consent for the use of data collected via questionnaires and clinics was obtained from participants following the recommendations of the ALSPAC Ethics and Law Committee at the time. Further information, including consent forms, can be found on the ALSPAC website (https://www.bristol.ac.uk/alspac/researchers/research‐ethics/). Consent is assumed to be implied by the provision of self‐report information and participants are free to withdraw their consent at any time. Separately, we obtained ethical approval for the present secondary data analysis from University College London's Institute of Education's Research Ethics Committee (REC1748). This study was not preregistered.

### Measures

#### Parental negativity

The main carer reported on PNeg at ages 4, 7 and 8 using items from a published scale (Dunn et al., 1998; Oliver & Pike, 2018) with established face validity and predictive validity (Dunn et al., 1998). The items capture evidence‐based aspects of parent negativity (e.g., lack of enjoyment, lack of acceptance and rejection). The three items were rated 1 (*yes*) or 0 (*no*): ‘I dislike the mess and noise that surrounds this child’, ‘I have frequent battles of will with this child’ and ‘This child gets on my nerves’. The internal consistency (Kuder & Richardson, 1937) values are low: 0.45, 0.55 and 0.56 for ages 4, 7 and 8, respectively.

#### Child externalising behaviour

Main‐carer reports from the Strengths and Difficulties Questionnaire (SDQ; Goodman, 1997) were used to measure child externalising behaviour at ages 4, 7 and 8 years. Items were rated as 0 (*not at all true*), 1 (*somewhat true*) and 2 (*certainly true*). We used a sum score for each the five‐item conduct problem and hyperactivity/inattention subscales and modelled these two scores as indicators of an externalising behaviour latent variable in the main analyses. Therefore, we captured the distinct aspects of conduct problems and of hyperactivity/inattention whilst also recognising that they are both externalising problems. Doing so provided the best fit to our data. Goodman et al. (2010) suggested that a model with the SDQ subscales as first order factors and with second order externalising and internalising problem factors is preferred in community low‐risk samples. A higher problem score indicates more externalising problems. The internal consistency (*α*) values were low for conduct problems (*α* = 0.51, 0.56 and 0.58 for ages 4, 7 and 8, respectively) and adequate for hyperactivity/inattention (*α* = 0.76, 0.77 and 0.80 for ages 4, 7 and 8, respectively).

#### Interpersonal social support

Main carers’ perceived interpersonal social support was reported when the child was around 5 years old. This measure has been used in previous studies using ALSPAC data and has shown good internal consistency and validity (e.g., Lähdepuro et al., 2023; Roulstone et al., 2011; Thomson et al., 2014; Tracy et al., 2018). It has seven items (e.g., ‘I have no one to share my feelings with’, ‘There are other [main carers] with whom I can share my experience’; see Table S3 for item list) rated on a four‐point response scale from 0 (*this is exactly how I feel*) to 3 (*I never feel this way*). Positively worded items were reverse‐coded, so that higher values reflected greater perceived interpersonal social support. Following other studies (e.g., Tracy et al., 2018), partner‐related items were recoded as *never feel this way* for parents without a partner (Table S3). A continuous sum score (*α* = 0.74) was created for each participant answering at least five out of the seven items. Then we created a binary variable reflecting the median split (median = 3.14) where group 1 = high interpersonal social support and group 2 = low interpersonal social support. Using a median split allowed us to conduct moderation models comparing the cross‐lagged model results of these two groups.

#### Neighbourhood social cohesion

Perceived neighbourhood social cohesion was measured with six items reported by the main carer when the child was around 5 years old. This measure has shown good reliability across developmental phases and construct validity in previous studies (Solmi et al., 2017). The measure is comprised of three questions relating to the main carer's neighbours (e.g., ‘Do the other people in your neighbourhood visit your home?’) and three items based on the main carer (e.g., ‘Do you look after your neighbour's children?’; see Table S3 for item list). Questions were rated on a 5‐point frequency scale from 1 (*No, never*) to 5 (*Almost every day*). The two items relating to lower cohesion (e.g., ‘keeping to oneself’) were reverse‐coded so that higher values indicated greater neighbourhood social cohesion. A continuous sum score was calculated if participants answered at least four out of the six items (*α* = 0.86). A binary variable was then created using a median split (median = 2.66) where group 1 = high neighbourhood cohesion and group 2 = low neighbourhood cohesion.

#### Covariates

We conditioned the second (age 7) and third timepoint (age 8) within‐person latent factors on child's sex assigned at birth. Child sex has been shown to relate to child behaviour differentially across ages (Bongers et al., 2004; Marçal, 2020) which violates the assumption of a Random‐Intercept Cross‐Lagged Panel Model (RI‐CLPM) that time invariant covariates have time‐fixed effects (Mund et al., 2021).

We also conditioned on several binary indicators of family adversity (see Measures section in Supporting Information S1) to guard against potential bias associated with non‐response. We used eight items from the Family Adversity Index (FAI; Steer & Wolke, 2004) developed by the ALSPAC team, covering a range of adversities reported by families at multiple waves during pregnancy through to when the child was aged 4 years. These items captured risks related to early parenthood, housing adequacy, housing (basic living), low education of mother and/or father, financial difficulties, partner status, maternal psychopathology and criminal behaviour. Each adversity item was assigned a value of 1 (present) or 0 (not present). These items are well known risk factors for child behavioural problems and PNeg and also showed statistically significant independent associations with missingness in a logistic regression model. These eight items were introduced as baseline covariates. The highest tetrachoric correlation between our adversity items was 0.4 and therefore we were unconcerned about multicollinearity, justifying entering them as separate independent variables in our models.

### Data analysis

**FIGURE 1:**
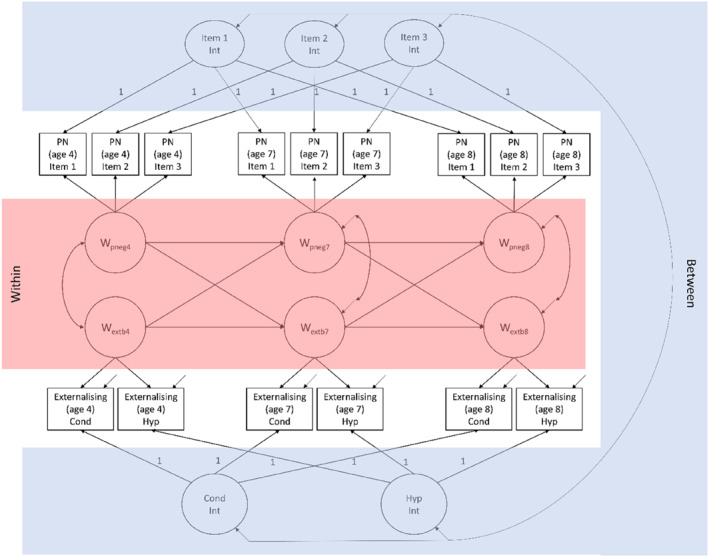
Schematic depiction of the multiple indicator random‐intercept cross‐lagged panel model for parental negativity (PNeg) and child externalising behaviours across ages 4, 7 and 8. Subscript numbers indicate the time point of assessment. Between‐person level of the Random‐Intercept Cross‐Lagged Panel Models model is indicated in grey. Fixed parameters are shown. Cross‐lagged paths were not constrained to be equal across time. The central curved arrows represent a within‐person correlation for the age 4 time point and within‐person residual correlations at the two subsequent time points (age 7 and 8). Cond, conduct problems; Hyp, hyperactivity/inattention; Int, intercept; PN, parental negativity; Wext, within‐person externalising behaviour; Wpneg, within‐person parental negativity.

Descriptive analyses were conducted in Stata MP (v 18.5), and SEM models estimated in Mplus (v 8.10; Muthén & Muthén, 1998–2017). To disaggregate within‐ from between‐person effects, as is recommended to address issues of ecological fallacy (Curran & Bauer, 2011; Mulder & Hamaker, 2021), we fitted RI‐CLPM to assess bidirectional associations between PNeg and child externalising behaviour across ages 4, 7 and 8 years (Figure 1). Disaggregating within‐ and between‐person sources of variability is considered important when studying stability and change in two or more concurrent processes (Curran & Bauer, 2011; Hamaker et al., 2015) and means that both cross‐lagged and autoregressive effects represent pairwise associations among the within‐person residuals and not the raw variables themselves.

To assess the robustness of our findings, we estimated Model 1 in a stepwise fashion starting with an unadjusted model, then a model adjusted for child sex and finally a model further adjusted for child sex and family adversity. This sequential modelling approach allowed us to assess the stability of the observed effects and to isolate the unique contributions of the primary variables. Our models were multiple indicator RI‐CLPMs (Figure 1). The pair of continuous SDQ sum‐scores were used as indicators of the externalising latent variables, and the three binary items of PNeg were used as indicators of PNeg. As such, an intercept was required for each manifest variable. The random intercepts represent the stable, between‐person ‘trait‐like’ influences across time for PNeg and child externalising behaviour. Additionally, a requirement for the multiple indicator RI‐CLPM is that item loadings are longitudinally invariant, that is there is (at least) weak factorial invariance over time (Mulder & Hamaker, 2021) (see Table S1 for additional information on measurement invariance testing).

The cross‐lagged paths (e.g., paths from ‘Wpneg’ and ‘Wextb’ shown in Figure 1) represent the effects of the within‐person deviation from their expected mean for PNeg and child externalising behaviour. As such, a positive cross‐lagged association between externalising behaviour at time *t* and PNeg at time *t* + 1 implies that a higher‐than‐average level of externalising behaviour for an individual is typically followed by a higher‐than‐average level of negativity for an individual (Curran & Bauer, 2011; Hamaker et al., 2015). The autoregressive paths (e.g., paths from ‘Wpneg4’ to ‘Wpneg7’ in Figure 1) represent the within‐person carry‐over effects from the earlier time point to the later time point (Speyer, Ushakova, et al., 2022). Whilst conceptually akin to quantifying stability in each of the two processes, the stable elements are instead modelled by the between‐person random intercepts and are distinct from the dynamic within‐person phenomena. The autoregressive and cross‐lagged unstandardised (and standardised where needed to aid in interpretation of effect size) parameter estimates are presented.

We further fitted two moderation models, one for each moderator (Models 2 and 3) which were fully adjusted. Specifically, we examined whether between‐person differences in perceived interpersonal social support at baseline (61 months) moderated cross‐lagged effects from PNeg to behavioural problems and vice versa (Model 2). A multiple‐group/moderation model defined by a median split (group 1 = high perceived interpersonal social support and group 2 = low perceived interpersonal social support) was used. Differences between groups were tested with Wald tests performed using Mplus' ‘model test’ command. If the Wald test *p* value was less than .05, we interpreted this as a lack of difference in any of the cross‐lagged effects across the three waves between the high and low social support groups. We subsequently tested the moderating effect of neighbourhood cohesion using a separate multiple‐group model (Model 3) again defined by a median split (group 1 = high perceived neighbourhood cohesion and group 2 = low perceived neighbourhood cohesion). For additional information on our moderation models, see Supporting Information S1.

#### Model fit

Model fit was evaluated by examining the Comparative Fit Index (CFI), Tucker‐Lewis Index, Root Mean Square Error of Approximation (RMSEA) and Standardised Root Mean Square Residual (SRMR; see Table 1). The recommended cut‐offs were CFI (≥0.95), SRMR (≤0.08), and RMSEA (≤0.06) (Hu & Bentler, 1999) meaning values close to these cut‐offs would indicate adequate fit of the model to the data.

**TABLE 1:** Means and standard deviations of the main variables in the analytic sample (*n* = 9943).

Variable	*N*	Mean (SD)
Externalising behaviour		
Conduct age 4	9123	1.96 (1.41)
Hyperactivity age 4	9046	3.96 (2.32)
Conduct age 7	7822	1.57 (1.44)
Hyperactivity age 7	7666	3.38 (2.37)
Conduct age 8	7246	1.49 (1.45)
Hyperactivity age 8	7240	3.33 (2.46)
Parental negativity		
Age 4	9111	0.96 (0.89)
Age 7	7781	0.70 (0.88)
Age 8	7767	0.70 (0.88)

*Note*: The sum scores presented for parental negativity here are for descriptive purposes and are not the values in the main measurement model.

#### Missing data

It is common practice when working with latent variables in a Structural Equation Modelling (SEM) framework to employ an approach that accommodates partial missingness among dependent variables, rather than using Multiple Imputation. Whilst both Maximum Likelihood (ML) and Multiple Imputation are based on the MAR‐assumption (Missing at Random), with the former method being generally simpler and more efficient for SEM, ML is often not feasible when working with categorical latent variable indicators due to the need for numerical integration. For our models, to permit the inclusion of the binary latent‐variable indicators for negative parenting, we instead used the WLSMV estimator (weighted least squares mean and variance adjusted) and the default theta parameterization. This is computationally less demanding than ML, as it makes use of polychoric correlations. However, the assumptions relating to missingness are weaker than for ML/imputation and potentially less tenable. WLSMV allows missingness to be only a function of observed covariates and not observed outcomes (Asparouhov & Muthén, 2006) and is sometimes referred to as ‘covariate‐dependent missing completely at random (MCAR)’. Given our substantive model only contains a single covariate—child's sex—we introduced further baseline data on family adversity to strengthen our missing data assumptions. Specifically, our family adversity variables were introduced as baseline covariates, and we regressed all five random intercepts on these variables. We note that, as with all approaches to missing data, our chosen method is reliant on untestable assumptions. However, we believe these assumptions are more justifiable following the inclusion of the adversity data as described.

## RESULTS

Descriptive statistics (Table 1) shows that conduct problems, hyperactivity and PNeg mean scores reduced slightly from ages 4 to 8 (statistics for the complete case sample is in Table S4). Moderate stability across ages 4, 7 and 8 is shown for conduct problems and hyperactivity and moderate‐to‐strong correlations were shown between PNeg across ages 4, 7 and 8. Conduct problems and PNeg were also moderately‐to‐strongly associated over time (Table 2). However, neighbourhood social cohesion and social support were weakly associated with externalising behaviours and PNeg. Additionally, boys showed more externalising behaviour overall compared to girls (see Table S5). See Tables S6 and S7 for the mean scores for child externalising behaviour and PNeg by high and low social support and neighbourhood cohesion. In line with our expectations, families in the low social support group and in the low neighbourhood cohesion group (relative to their high social support and cohesion group counterparts) showed higher average conduct and hyperactivity problems and higher average PNeg.

**TABLE 2:** Correlation matrix for main study variables.

		1	2	3	4	5	6	7	8	9	10	11
1	Conduct age 4	1										
2	Hyperactivity age 4	0.47	1									
3	Parental negativity age 4	0.56	0.39	1								
4	Conduct age 7	0.49	0.34	0.39	1							
5	Hyperactivity age 7	0.35	0.57	0.31	0.46	1						
6	Parental negativity age 7	0.43	0.33	0.60	0.50	0.39	1					
7	Conduct age 8	0.50	0.34	0.41	0.62	0.39	0.52	1				
8	Hyperactivity age 8	0.35	0.55	0.31	0.40	0.73	0.39	0.49	1			
9	Parental negativity age 8	0.42	0.32	0.61	0.49	0.38	0.74	0.63	0.46	1		
10	Social support age 5	−0.16	−0.14	−0.13	−0.15	−0.12	−0.14	−0.16	−0.14	−0.11	1	
11	Neighbourhood cohesion age 5	−0.05	−0.07	−0.03	−0.03	−0.05	−0.06	−0.07	−0.04	−0.04	0.35	1

*Note*: The polychoric correlation command in Stata was used (Kolenikov, 2016) which chooses the appropriate approach depending on the variable for example, Pearson correlation for continuous, tetrachoric for a pair of binary variables.

### Autoregressive and cross‐lagged effects within the main model (Model 1)

The positive autoregressive paths for both PNeg and child externalising problems, from age 4 to age 7 and from age 7 to age 8, indicated moderate stability across ages especially between ages 7 and 8 (Table 3; *β*s ranging 0.53 to 0.76). The cross‐lagged effects in the main‐group model revealed that within‐person change in PNeg at age 7 predicted within‐person change in child externalising behaviour at age 8 (Table 3; *β* = 0.15). That is, when the main carer reported higher than their usual negativity when their child was 7, their child's externalising behaviour at age 8 was also higher than their child's usual level of externalising behaviour. The same was true for the child‐to‐parent relationship from ages 7 to 8 (*β* = 0.13): within‐person change in children's externalising behaviour at age 7 predicted within‐person change in PNeg at age 8. In other words, when children's externalising behaviour at age 7 was higher than their usual level, PNeg at age 8 was also higher than the main carer's usual level. As one would expect, the autoregressive and cross‐lagged paths were stronger between age 7 and age 8 given they are closer in time (Table 3). No bidirectional associations within families were found between PNeg and child externalising behaviours from ages 4 to 7 (*β*s <0.03). Results for the complete case sample and fully adjusted model are reported in Tables S8 and S9.

**TABLE 3:** Results for the RI‐CLPMs for the main model using the available case sample (*n* = 9943).

		Unconditional				Condition on child sex				Condition on child sex, plus family adversities			
		*b* (SE)	*p*	95% CI	*β*	*b* (SE)	*p*	95% CI	*β*	*b* (SE)	*p*	95% CI	*β*
Cross‐lagged effects													
PNeg → Ext	4 years → 7 years	0.15 (0.174)	.376	−0.191 to 0.491	0.13	0.06 (0.139)	.655	−0.212 to 0.332	0.06	0.01 (0.129)	.933	−0.243 to 0.263	0.01
	7 years → 8 years	0.27 (0.062)	<.001	0.148–0.392	0.21	0.19 (0.053)	<.001	0.086–0.294	0.17	0.16 (0.048)	.001	0.066–0.254	0.15
Ext → PNeg	4 years → 7 years	0.07 (0.086)	.451	−0.099 to 0.239	0.07	0.08 (0.081)	.311	−0.079 to 0.239	0.08	0.03 (0.080)	.698	−0.127 to 0.187	0.03
	7 years → 8 years	0.13 (0.042)	.002	0.048–0.212	0.15	0.13 (0.047)	.005	0.038–0.222	0.14	0.12 (0.046)	.007	0.030–0.210	0.13
Autoregressive effects													
PNeg → PNeg	4 years → 7 years	0.62 (0.127)	<.001	0.371–0.869	0.61	0.62 (0.114)	<.001	0.397–0.843	0.60	0.61 (0.113)	<.001	0.389–0.831	0.60
	7 years → 8 years	0.78 (0.054)	<.001	0.674–0.886	0.76	0.79 (0.053)	<.001	0.686–0.894	0.77	0.78 (0.053)	<.001	0.676–0.884	0.76
Ext → Ext	4 years → 7 years	0.49 (0.170)	.004	0.157–0.823	0.48	0.58 (0.158)	<.001	0.270–0.890	0.56	0.56 (0.155)	<.001	0.256–0.864	0.53
	7 years → 8 years	0.75 (0.062)	<.001	0.628–0.872	0.070	0.81 (0.062)	<.001	0.688–0.932	0.76	0.80 (0.058)	<.001	0.686–0.914	0.76
Fit statistics													
CFI		0.994				.985				0.985			
TLI		0.990				.977				0.978			
RMSEA		0.020				.029				0.021			

*Note*: Unstandardized coefficients for cross‐lagged and autoregressive effects are reported followed by standard errors in parentheses. Standardised coefficients are indicated by *β*.

Abbreviations: Ext, externalising behaviour; PNeg, parental negativity.

### Cross‐lagged effects within the moderation models (Models 2 and 3)

Table 4 shows results from our two multiple‐group models stratified by high/low interpersonal social support and high/low neighbourhood cohesion. We focus on the evidence for differences between groups of the cross‐lagged effects, as these are the paths relevant to our hypotheses. Differences across strata for these cross‐lagged parameters were calculated, along with standard errors (derived using the delta method), and accompanying estimated Wald statistics and *p‐*values, quantifying evidence for moderation of cross‐lagged paths in either direction (Table 4). We did not find evidence of moderation of the cross‐lagged paths from PNeg to externalising behaviour by either interpersonal social support or neighbourhood cohesion. We also did not find evidence of moderation of the paths from externalising behaviour to PNeg.

**TABLE 4:** Results for the RI‐CLPMs for models testing moderation by interpersonal social support (Model 2) and neighbourhood cohesion (Model 3) in separate models adjusted for sex and family adversity.

		Interpersonal social support (Model 2)				Neighbourhood cohesion (Model 3)			
		Low (*n* = 3698)	High (*n* = 4684)	Differences across strata	95% CI	Low (*n* = 4140)	High (*n* = 4215)	Differences across strata	95% CI
		*b* (SE)	*b* (SE)	*b* (SE)		*b* (SE)	*b* (SE)	*b* (SE)	
Cross‐lagged effects									
PNeg → Ext	4 years → 7 years	−0.070 (0.336)	−0.050 (0.197)	−0.020 (0.317)	−0.641 to 0.601	−0.086 (0.164)	−0.012 (0.218)	−0.075 (0.191)	−0.439 to 0.415
	7 years → 8 years	0.093 (0.097)	0.277 (0.074)	−0.184 (0.120)	−0.419 to 0.05	0.266 (0.062)	0.072 (0.083)	0.194 (0.098)	−0.09 to 0.234
				Wald = 2.38, *p* = .305				Wald = 3.97, *p* = .134	
Ext → PNeg	4 years → 7 years	0.317 (0.257)	0.113 (0.131)	0.203 (0.257)	−0.30 to 0.706	0.139 (0.134)	0.234 (0.187)	−0.095 (0.183)	−0.132 to 0.6
	7 years → 8 years	0.249 (0.072)	0.195 (0.051)	0.055 (0.083)	−0.107 to 0.217	0.201 (0.055)	0.261 (0.081)	−0.060 (0.088)	0.102–0.419
				Wald = 1.02, *p* = .602				Wald = 0.70, *p* = .706	

*Note*: Unstandardized coefficients for cross‐lagged effects are reported followed by standard errors in parentheses. Ext = externalising behaviour; PNeg = parental negativity. Differences and their standard errors derived using Mplus' ‘model constraint’ and 2 d.f. Wald tests performed using Mplus' ‘model test’ command.

## DISCUSSION

To our knowledge, for the first time, we used a large UK cohort sample (ALSPAC) to examine whether perceived interpersonal social support from family, friends and neighbours and neighbourhood social cohesion helped to break negative parent–child cycles. We modelled within‐person associations between PNeg and child externalising behaviour across ages 4, 7 and 8, finding bidirectional (parent and child) within‐person effects from ages 7 to 8 only. Contrary to expectations, we found no evidence of differences in these associations as a function of interpersonal social support and neighbourhood social cohesion.

Our identification of bidirectional parent–child processes using RI‐CLPM in middle childhood provides support for Patterson's coercion model, which theorises about the within‐dyad effects in a family (Patterson, 1992; Speyer, Hang, et al., 2022). Based on guidance for interpreting these effects (Orth et al., 2024), we consider our cross‐lagged findings in our main model from ages 7 to 8 to be large (Orth et al., 2024 suggests that standardised effect sizes of 0.12 and above are large), although the lack of these effects from ages 4 to 7 was contrary to our expectations. Given we found less stability in our autoregressive paths between ages 4 and 7 compared to ages 7 and 8, we speculate that this is most likely due to the larger time lag at this stage of the model which can influence the size of cross‐lagged effects (Hamaker, 2023; Orth et al., 2024). Bidirectional processes between parents and children occur both over time (macro‐level) and moment‐to‐moment (micro‐level). Macro‐level assessments over many years like the ones modelled here capture important parent–child processes but offer only a limited picture of parent–child relationships. Moreover, the larger the gap between assessments, the less able we are to understand potential changes in the patterns of mutual influences between parents and children. Specifically, developmental changes in both the children and in parenting can take place during these periods that would not be captured in these models to complete the picture of bidirectionality within parent–child dyads. Our hope is that the increasingly open nature of our science allows researchers to share data and thus unpick macro‐ and micro‐processes in large samples with well‐defined and detailed assessments.

The lack of evidence for moderation by interpersonal social support was not expected, since social support is a known determinant of parenting (Belsky, 1984; Taraban & Shaw, 2018) and was considered a reasonable candidate for moderating the parent–child coercive cycle. One possibility is that social support does not influence parent–child processes in the same way (or direction) for all families, which would not be captured by our analyses. That is, although support for parents is generally considered to be a positive influence, by nature of using perceptions of support, high‐quality social support may not necessarily equate to positive influences on the parent–child processes. There is some research indicating that social support can be associated with negative parenting (Driscoll & Easterbrooks, 2007). For example, perceptions of a high‐quality coparenting‐partnership— one aspect of interpersonal social support — has been associated with increased externalising behaviours in children in family contexts where maternal parenting is characterised by coerciveness or harshness (Latham et al., 2018). Alternatively, interpersonal social support may be important for reducing PNeg and children's externalising behaviour in isolation but may not be as important for the parent–child processes that occur during early‐to‐middle childhood. Future research considering a role for perceptions of social support alongside objective measures of support for the parent–child coercive process would be of great interest.

We also expected to find that the neighbourhood context could act as a buffer. We hypothesised that cohesive neighbourhoods would have wider social networks available to parents and children, providing non‐parental opportunities for monitoring and looking after children (Heberle et al., 2015), particularly given the literature showing that neighbourhood cohesion factors might help protect against mental‐health problems related to family risk including socio‐economic disadvantage and child maltreatment (Abdullah et al., 2020; Kingsbury et al., 2015). For example, a systematic review (Abdullah et al., 2020) found that neighbourhood collective efficacy, individuals' perception of closeness or connection with their neighbours (of which social cohesion is part) and the capacity of neighbours to intervene on behalf of their community to reach common goals (Sampson et al., 1997), acts as a buffer for child maltreatment (psychological aggression and physical assault). However, we did not find evidence of neighbourhood social cohesion as a moderator of parent–child bidirectional associations. We suspect that the role of neighbourhood cohesion (and interpersonal social support) in the association between PNeg and child behaviour is quite complex. For example, differences in the child‐on‐parent effects may not be captured here through parent reports and/or through a measure of the parents' interpersonal social support (rather than the child's). Therefore, the social support and community experience of the child may be overlooked. Moreover, there may be other factors such as child temperament and parent personality that may confound these associations. For example, those who are more sociable may be more likely to receive social support and may also contribute, albeit indirectly, to the social cohesion in their area.

Additionally, our study examined bidirectional within‐individual processes over time which differs from those of the wider literature exploring neighbourhood social context (and interpersonal social support) as moderators. First, these studies typically are not bidirectional and instead examine directional relationships between parenting (the independent variable) and child behaviour (the dependent variable). With regard to the parent‐on‐child effects, our findings are contrary to existing findings showing that the social context including neighbourhood social cohesion can protect children from poor parenting risk (Abdullah et al., 2020; Kingsbury et al., 2015). As for the path from child‐to‐parent, there are no studies to our knowledge which have explored moderation by neighbourhood context. Second, in the RI‐CLPM, the stable, between‐person variance is separated from the within‐person variance leading to the cross‐lagged effects only capturing within‐person carry‐over. This is contrary to studies examining moderation of between‐person prospective effects. We expect that moderation by social environment may be more pertinent to the trait‐like/between‐person associations captured by the random‐intercept than the within‐person associations.

Our study has important strengths, including the novel exploration of moderation of parent–child processes over several years in a general population sample and using RI‐CLPM to disaggregate between‐ and within‐person influences. Such models align well with Patterson's (1992) suggested within‐person changes reflected in coercive cycles within the family. However, we acknowledge several limitations. First, our secondary analysis is restricted by the data available, and we were only able to model three uneven timepoints which make the interpretation of our results more challenging. For example, the time windows between questionnaire waves may be too short, or too long, to capture the reciprocal effects we expected to see, and we are not able to accurately capture developmental trajectories. Second, our measure of parenting captures feelings of hostility and negativity but does not include important negative parenting facets––especially harsh discipline tactics (e.g., shouting, smacking) –– likely to be important. Moreover, the low internal consistency of the PNeg items suggests this measure may not be reliable. Third, self‐reported measures are sensitive to common‐method bias, where personality or affective traits influence individuals' evaluations of the experiences of their family (Meltzer et al., 2007). Parents' expectations of their specific child's behaviour also might play a role since parents who perceive their child as difficult, hyperactive or disruptive may be more negatively disproportionately reactive to their child (Ansari & Crosnoe, 2016). Fourth, although we adjusted for a range of family adversities experienced throughout early childhood, we did not account for time‐varying changes in family experiences across ages 4 to 8. Future work better able to consider these limitations and considering a combination of objective and subjective measures would be valuable.

## CONCLUSION

We hope that our study will prompt research considering moderation of longitudinal parent–child processes in community samples. Ultimately, understanding the complex processes that unfold within families and the role of the social environment in potentially breaking negative cycles may help us identify additional targets supporting family relationships and wellbeing.

## AUTHOR CONTRIBUTIONS

**Jasmine A. L. Raw**: Conceptualization; data curation; formal analysis; methodology; project administration; visualization; writing—original draft; writing—review and editing. **Jon Heron**: Formal analysis; methodology; supervision; visualization; writing—original draft; writing—review and editing. **Bonamy R. Oliver:** Conceptualization; funding acquisition; methodology; supervision; writing—original draft; writing—review and editing. **Jane Gilmour**: Conceptualization; writing—review and editing. **Emily Midouhas**: Conceptualization; funding acquisition; methodology; project administration; supervision; writing—original draft; writing—review and editing.

## CONFLICT OF INTEREST STATEMENT

The authors declare no conflicts of interest.

## ETHICAL CONSIDERATIONS

Ethical approval for the study was obtained from the ALSPAC Law and Ethics Committee (IRB00003312) on 28 of June 1990. We also obtained ethical approval for the present study from UCL IOEs Research Ethics Committee on 9 January 2023 (REC1748). Informed consent for the use of data collected via questionnaires and clinics was obtained from participants following the recommendations of the ALSPAC Ethics and Law Committee at the time. Further information, including consent forms can be found on the ALSPAC website (https://www.bristol.ac.uk/alspac/researchers/research‐ethics/).

## Supporting information

Supporting Information S1

## Data Availability

Access to ALSPAC data is through a system of managed open access (http://www.bristol.ac.uk/alspac/researchers/access/).
