# Supplementary material for: Bidirectional associations between parental negativity and child externalising problems: Social support and neighbourhood cohesion as moderators
Source: JCPP Adv. 2025 Oct 8;6(2):e70054. doi: 10.1002/jcv2.70054 (PMC13260671; doi:10.1002/jcv2.70054)
Supplement: Supplementary file 1 — Supporting Information S1 [file JCV2-6-e70054-s001.docx]

**Supporting Information**


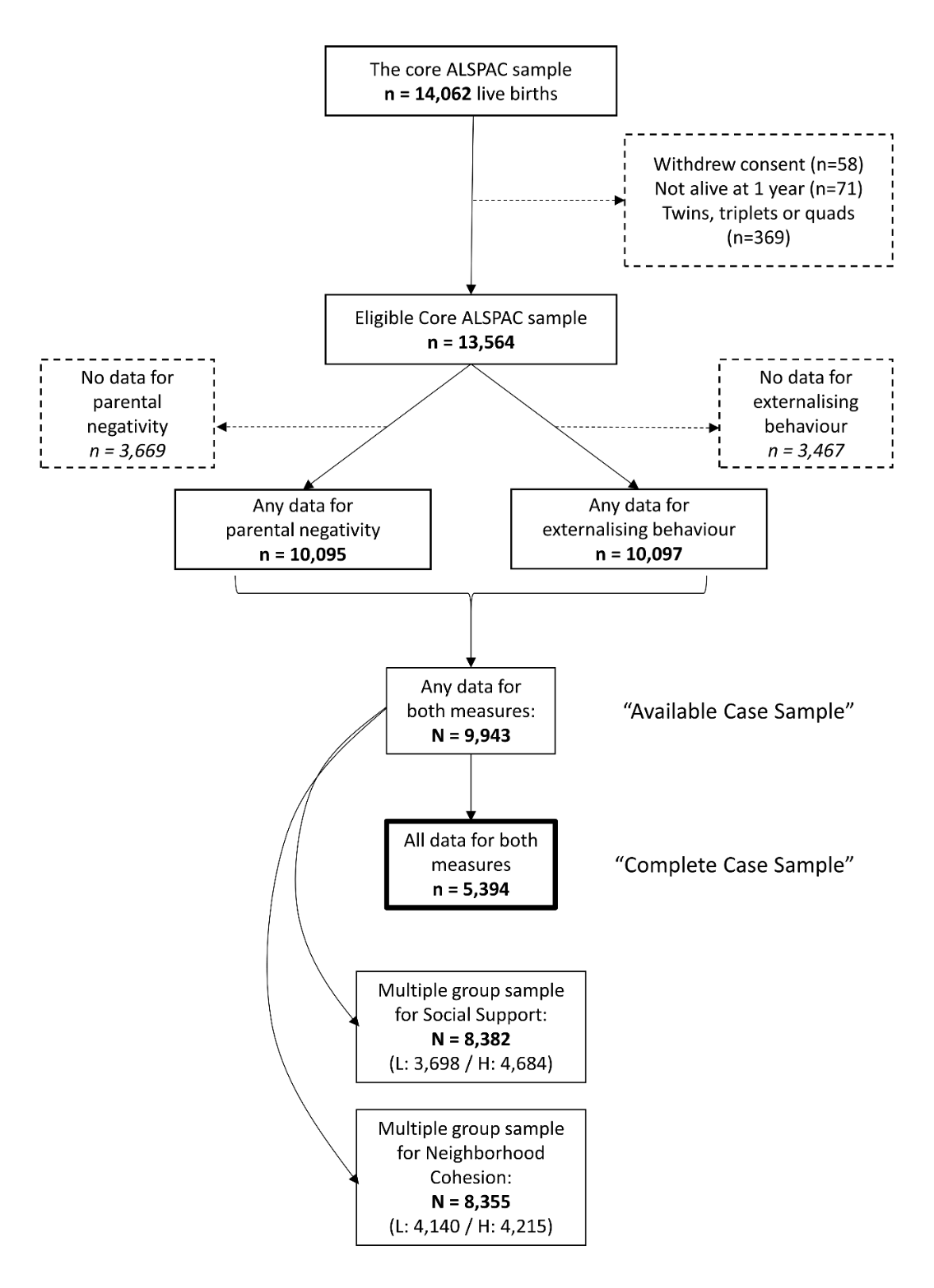
***Figure S1. Flowchart of attrition***

**Measures**

***Family Adversity***

We measured family adversity with the Family Adversity Index (FAI; Collin et al., 2015), an ALSPAC-derived measure which consists of 18 adversity items that were collected at three distinct time periods: pregnancy, 0-2 years and 2-4 years. Each item was binary (1 = adversity occurred, 0 = adversity did not occur). Missing values were given a zero (i.e., indicating the adversity did not occur) - this was relevant only in less than 1% of the sample for each adversity. Then we selected those that were found to be independently predictive of whether the family had provided a complete or incomplete set of the dependent variables required for our models in a logistic regression model. The eight items selected based on the model were: 1) early parenthood, 2) housing adequacy, 3) housing (basic living), 4) low education of mother and/or father, 5) financial difficulties, 6) partner status, 7) maternal psychopathology and 8) criminal behaviour. These were entered as eight binary variables in the main models.

**Data Analysis**

***Moderation models***

As a supplement to their paper, Mulder and Hamaker (2021) have produced a useful web resource: https://jeroendmulder.github.io/RI-CLPM/ which describes the code required to estimate a series of RI-CLPM models using Mplus. We followed their guidance in terms of the constraints, and relaxed constraints, needed for estimating a multiple-group model. This resource does not extend to a multiple-group multiple-item RI-CLPM, so we adapted their guidance to accommodate the latent variable indicators. All latent variable means/intercepts were constrained to zero in the second group, the means/thresholds for the latent variable indicators were freely estimated and allowed to differ from the first group, and associations between latent variables and both sex and, when relevant, the indicators of family adversity, were also allowed to vary between groups.

***Measurement invariance***

A requirement for the multiple indicator RI-CLPM is that item loadings are longitudinally invariant, i.e. there is (at least) weak factorial invariance over time (Mulder & Hamaker, 2021). To examine evidence for measurement variance we separately estimated a series of models for parental negativity and then for externalising behaviour, taking the stepwise approach described by Lesa Hoffman (Hoffman, 2015). The within-person component of our final chosen model was time-invariant for the factor loadings apart from the ‘dislike of mess’ item in the parental negativity scale) at age 4 where the loading was lower.

A pair of models were estimated with item loadings initially freed and then constrained to be longitudinally invariant. The “difftest” command in Mplus (Asparouhov & Muthén, 2006), used for testing between nested models which have been estimated using WLSMV, was used to compare these two models. If the difftest p-value showed the invariant model to be inferior we used modification indices as a guide and sequentially removed constraints for individual loadings, repeating the difftest step as we moved incrementally towards the fully free model. This resulted in models for which we had loadings that were partially longitudinally invariant (See Hoffman).

The model comparisons for negative parenting are shown in Table S10. Each item in turn was selected to have its loadings fixed to be equal to 1 (i.e. to fix the scale of the latent variable). The time-invariant model, with all loadings constrained to be equal across wave, was found to have substantially poorer fit when compared to the fully non-invariant model (χ^2^= 98.35 , p < 0.001). However a partially invariant model in which the loading for “dislike of mess” was freely estimated, was found to fit as well as the fully non-invariant model (χ^2^ = 3.38 , p = 0.337). Carrying out the same steps for externalising problems showed the time-invariant model to be acceptable (χ^2^= 3.986, p = 0.13

**Table S1**

| *Using difftest to obtain a model with partial measurement invariance for the within-subject negative parenting latent variable* | Battle of wills has fixed loading | | |  | Dislike of mess has fixed loading | | |  | Child gets on nerves has fixed loading | | |
| --- | --- | --- | --- | --- | --- | --- | --- | --- | --- | --- | --- |
|  | Time-varying loading model | Time-invariant loading model | Partially invariant-loading model |  | Time-varying loading model | Time-invariant loading model | Partially invariant-loading model |  | Time-varying loading model | Time-invariant loading model | Partially invariant-loading model |
|  |  |  |  |  |  |  |  |  |  |  |  |
| *W_neg4* |  |  |  |  |  |  |  |  |  |  |  |
| Wills λ | 1.000 | 1.000 | 1.000 |  | 1.610 | 0.750 | 1.028 |  | 0.964 | 1.086 | 1.042 |
| Mess λ | 0.633 | 1.333 | *0.593 |  | 1.000 | 1.000 | *0.609 |  | 0.608 | 1.448 | *0.618 |
| Nerves λ | 1.039 | 0.921 | 0.959 |  | 1.672 | 0.691 | 0.986 |  | 1.000 | 1.000 | 1.000 |
|  |  |  |  |  |  |  |  |  |  |  |  |
| *W_neg7* |  |  |  |  |  |  |  |  |  |  |  |
| Wills λ | 1.000 | 1.000 | 1.000 |  | 1.021 | 0.750 | 1.028 |  | 0.989 | 1.086 | 1.042 |
| Mess λ | 0.980 | 1.333 | 0.972 |  | 1.000 | 1.000 | 1.000 |  | 0.969 | 1.448 | 1.013 |
| Nerves λ | 1.012 | 0.921 | 0.959 |  | 1.032 | 0.691 | 0.986 |  | 1.000 | 1.000 | 1.000 |
|  |  |  |  |  |  |  |  |  |  |  |  |
| *W_neg8* |  |  |  |  |  |  |  |  |  |  |  |
| Wills λ | 1.000 | 1.000 | 1.000 |  | 0.955 | 0.750 | 1.028 |  | 1.025 | 1.086 | 1.042 |
| Mess λ | 1.004 | 1.333 | 0.972 |  | 1.000 | 1.000 | 1.000 |  | 1.029 | 1.448 | 1.013 |
| Nerves λ | 0.977 | 0.921 | 0.959 |  | 0.971 | 0.691 | 0.986 |  | 1.000 | 1.000 | 1.000 |
|  |  |  |  |  |  |  |  |  |  |  |  |
| DIFFTEST | - | χ^2^= 98.35  p < 0.001 | χ^2^ = 3.38  p = 0.337 |  |  | χ^2^ = 98.57  p < 0.001 | χ^2^ = 3.38  p = 0.338 |  |  | χ^2^ = 98.38  p < 0.001 | χ^2^ = 3.38  p = 0.337 |

**Table S2**

*Sample demographics for binary variables for the complete case and available case samples.*

|  | Complete case sample (*n* = 5394) | Available case sample (*n* = 9943) |
| --- | --- | --- |
| **Variable** | N (%) | N (%) |
| High social support* | 2,993/5,164 (58.0%) | 4,684/8,382 (55.9%) |
| High neighbourhood cohesion* | 2,700/5,144 (52.5%) | 4,215/8,355 (50.1%) |
| Child’s sex at birth (female) | 2,676 (49.6%) | 4,818 (48.5%) |
| *Family adversity* |  |  |
| Early parenthood | 201 (3.7%) | 571 (5.7%) |
| Housing (adequacy) | 645 (12.0%) | 1,498 (15.1%) |
| Housing (basic living) | 368 (6.8%) | 776 (7.8%) |
| Low education | 501 (9.3%) | 1,189 (12.0%) |
| Financial difficulties | 1,030 (19.1%) | 2,151 (21.6%) |
| Partner status | 749 (13.9%) | 1,581 (15.9%) |
| Maternal psychopathology | 1,759 (32.6%) | 3,555 (35.8%) |
| Criminal behaviour | 313 (5.8%) | 754 (7.6%) |

*Note.* *Denominator shown when lower than the stated sample size.

| Measure | Question and item wording | Response options | Notes |
| --- | --- | --- | --- |
| Perceived interpersonal social support | 1. I have no one to share my feelings with. | 1 – *This is exactly how I feel*.  2 – *This is often how I feel.*  3 – *This is how I sometimes feel.*  4 – *I never feel this way.*  7 – *Have no partner.*  (for items 2, 5, and 7) | Items 2-4, 6 and 7 were reverse coded so that higher values indicated more support. Response values of 7 on items 2 and 7 were recoded to a value of ‘1’. For item 5, a response of 7 was recoded to a value of ‘4’. The recoded variables were then averaged to create a social support score. |
|  | 2. My partner provides the emotional support I need. |  |  |
|  | 3. There are other mothers with whom I can share my experience. |  |  |
|  | 4. I believe in moments of difficulty my neighbours would help me. |  |  |
|  | 5. I’m worried that my partner might leave me. |  |  |
|  | 6. There is always someone with whom I can share my happiness and excitement about my child. |  |  |
|  | 7. If I feel tired I can rely on my partner to take over. |  |  |
| Perceived neighbourhood cohesion | *Do the other people in your neighbourhood…*   1. Visit your home? 2. Look after your children? 3. Keep to themselves | 1 – *No, never*  2 – *Rarely*  3 – *Sometimes*  4 – *Often*  5 – *Almost every day* | One item from the neighbour-related questions and one item from the mother-related questions will be reverse coded so that higher values indicate more perceived neighbourhood support |
|  | *Do you…*   1. Visit the home of your neighbours? 2. Look after your neighbour’s children? 3. Keep to yourself? |  |  |

**Table S3**

*Question items and response options for perceived interpersonal social support and neighbourhood cohesion*

**Table S4**

*Means and standard deviations of the main variables for the complete case sample*

|  | Complete case sample  (*n* = 5394) |
| --- | --- |
| *Variable* | Mean (SD) |
| Externalising behaviour |  |
| Conduct age 4 | 1.88 (1.38) |
| Hyperactivity age 4 | 3.83 (2.33) |
| Conduct age 7 | 1.52 (1.43) |
| Hyperactivity age 7 | 3.29 (2.36) |
| Conduct age 8 | 1.44 (1.42) |
| Hyperactivity age 8 | 3.24 (2.45) |
| Parental negativity |  |
| Negativity age 4 | 0.96 (0.89) |
| Negativity age 7 | 0.70 (0.88) |
| Negativity age 8 | 0.70 (0.88) |

*Note.* The sum-scores presented for parental negativity here are for descriptive purposes and are not the values in the main measurement model

**Table S5**

*Main variable descriptives by sex at birth for the complete case and available case samples*

|  |  | Complete case sample (*n* = 5,394) | | | | | Available case sample (*n* <= 9,943) | | | | | |
| --- | --- | --- | --- | --- | --- | --- | --- | --- | --- | --- | --- | --- |
|  |  | Boys | | Girls | |  | Boys | | Girls | |  |  |
| *Variable* | Age | N | Mean (SD) | N | Mean (SD) | Sex differences | N | Mean (SD) | N | Mean (SD) | Sex-difference | *p*-value |
| Conduct | 4 | 2,718 | 1.95 (1.41) | 2,676 | 1.81 (1.35) | -.137 [-.211, -.063] | 4,704 | 2.03 (1.44) | 4,419 | 1.88 (1.37) | -.146 [-.204, -.089] | < .001 |
|  | 7 | 2,718 | 1.59 (1.44) | 2,676 | 1.45 (1.42) | -.136 [-.212, -.060] | 3,998 | 1.65 (1.45) | 3,824 | 1.49 (1.42) | -.156 [-.220, -.092] | < .001 |
|  | 8 | 2,718 | 1.53 (1.46) | 2,676 | 1.35 (1.37) | -.184 [-.260, -.109] | 3,670 | 1.58 (1.50) | 3,576 | 1.39 (1.39) | -.199 [-.265, -.132] | < .001 |
| Hyperactivity | 4 | 2,718 | 4.12 (2.39) | 2,676 | 3.53 (2.22) | -.594 [-.717, -.471] | 4,664 | 4.25 (2.36) | 4,382 | 3.66 (2.24) | -.590 [-.685, -.495] | < .001 |
|  | 7 | 2,718 | 3.68 (2.48) | 2,676 | 2.90 (2.18) | -.781 [-.902, -.656] | 3,933 | 3.77 (2.47) | 3,733 | 2.96 (2.19) | -.814 [-.919, -.709] | < .001 |
|  | 8 | 2,718 | 3.69 (2.56) | 2,676 | 2.77 (2.23) | -.922 [-1.05, -.794] | 3,662 | 3.79 (2.57) | 3,578 | 2.85 (2.24) | -.930 [-1.04, -.819] | < .001 |
| Parental negativity | 4 | 2,718 | 0.95 (0.89) | 2,676 | 0.96 (0.89) | .012 [-.035, .060] | 4,695 | 0.97 (0.91) | 4,416 | 0.98 (0.90) | .002 [-.035, .039] | .910 |
|  | 7 | 2,718 | 0.69 (0.89) | 2,676 | 0.71 (0.87) | .028 [-.019, .075] | 3,989 | 0.71 (0.89) | 3,792 | 0.73 (0.89) | .025 [-.015, .064] | .221 |
|  | 8 | 2,718 | 0.69 (0.89) | 2,676 | 0.71 (0.87) | .015 [-.032, .062] | 3,678 | 0.71 (0.90) | 3,589 | 0.74 (0.88) | .021 [-.020, .062] | .313 |

**Table S6**

*Main variable descriptives by high and low social support and neighbourhood cohesion for the available case sample (n = 9,943).*

|  |  | Social support | |  | Neighbourhood cohesion | |  |
| --- | --- | --- | --- | --- | --- | --- | --- |
|  |  | Low (n <= 3,457) | High (n <= 4,446) |  | Low  (n <= 3,858) | High  (n <= 3,988) |  |
| Variable | Age | Mean (SD) | Mean (SD) | Difference | Mean (SD) | Mean (SD) | Difference |
| Conduct | 4 | 2.13 (0.03) | 1.80 (0.02) | -0.34 [-0.40, -0.28] | 1.99 (0.02) | 1.90 (0.02) | -0.09 [-0.15, -0.03] |
|  |  |  |  | *p < .001* |  |  | *p = .004* |
|  | 7 | 1.75 (0.03) | 1.43 (0.02) | -0.32 [-0.39, -0.26] | 1.62 (0.03) | 1.52 (0.02) | -0.09 [-0.16, -0.03] |
|  |  |  |  | *p < .001* |  |  | *p = .007* |
|  | 8 | 1.69 (0.03) | 1.32 (0.02) | -0.37 [-0.44, -0.30] | 1.55 (0.03) | 1.41 (0.02) | -0.15 [-0.22, -0.08] |
|  |  |  |  | *p < .001* |  |  | *p < .001* |
| Hyperactivity | 4 | 4.25 (0.04) | 3.70 (0.03) | -0.54 [-0.65, -0.44] | 4.09 (0.04) | 3.81 (0.04) | -0.28 [-0.38, -0.17] |
|  |  |  |  | *p < .001* |  |  | *p < .001* |
|  | 7 | 3.65 (0.04) | 3.16 (0.04) | -0.49 [-0.60, -0.38] | 3.49 (0.04) | 3.26 (0.04) | -0.24 [-0.35, -0.13] |
|  |  |  |  | *p < .001* |  |  | *p < .001* |
|  | 8 | 3.65 (0.05) | 3.05 (0.04) | -0.60 [-0.72, -0.48] | 3.41 (0.04) | 3.22 (0.04) | -0.1 [-0.31, -0.073] |
| Parental Negativity |  |  |  | *p < .001* |  |  | *p = .002* |
|  | 4 | 1.07 (0.02) | 0.91 (0.01) | -0.16 [-0.20, -0.12] | 1.00 (0.01) | 0.97 (0.01) | -0.03 [-0.07, 0.01] |
|  |  |  |  | *p < .001* |  |  | *p = .100* |
|  | 7 | 0.84 (0.02) | 0.64 (0.01) | -0.20 [-0.24, -0.16] | 0.77 (0.02) | 0.69 (0.01) | -0.08 [-0.12, -0.04] |
|  |  |  |  | *p < .001* |  |  | *p < .001* |
|  | 8 | 0.84 (0.02) | 0.64 (0.01) | -0.20 [-0.24, -0.15] | 0.76 (0.02) | 0.70 (0.01) | -0.06 [-0.11, -0.02] |
|  |  |  |  | *p < .001* |  |  | *p = .004* |

**Table S7**

*Main variable descriptives by high and low social support and neighbourhood cohesion for the complete case sample (n = 5,394)*

|  |  | Social support | |  | | Neighbourhood cohesion | | |  | |
| --- | --- | --- | --- | --- | --- | --- | --- | --- | --- | --- |
|  |  | Low  (n <=3,457) | High  (n <=4,446) |  | | Low (n <= 3,858) | High (n <= 3,988) |  | | |
| Variable | Age | Mean (SD) | Mean (SD) | Differences | | Mean (SD) | Mean (SD) | Differences | | |
| Conduct | 4 | 2.13 (0.03) | 1.80 (0.02) | -0.338 | [-0.401, -0.276] | 1.99 (0.02) | 1.90 (0.02) | -0.091 | | [-0.153, -0.028] |
|  | 7 | 1.75 (0.03) | 1.43 (0.02) | -0.323 | [-0.390, -0.256] | 1.62 (0.03) | 1.52 (0.02) | -0.093 | | [-0.159, -0.026] |
|  | 8 | 1.69 (0.03) | 1.32 (0.02) | -0.374 | [-0.444, -0.304] | 1.55 (0.03) | 1.41 (0.02) | -0.146 | | [-0.216, -0.076] |
| Hyperactivity | 4 | 4.25 (0.04) | 3.70 (0.03) | -0.542 | [-0.645, -0.439] | 4.09 (0.04) | 3.81 (0.04) | -0.277 | | [-0.380, -0.174] |
|  | 7 | 3.65 (0.04) | 3.16 (0.04) | -0.492 | [-0.603, -0.381] | 3.49 (0.04) | 3.26 (0.04) | -0.237 | | [-0.348, -0.126] |
|  | 8 | 3.65 (0.05) | 3.05 (0.04) | -0.602 | [-0.721, -0.484] | 3.41 (0.04) | 3.22 (0.04) | -0.192 | | [-0.311, -0.073] |
| Parental Negativity | 4 | 1.07 (0.02) | 0.91 (0.01) | -0.161 | [-0.201, -0.121] | 1.00 (0.01) | 0.97 (0.01) | -0.033 | | [-0.073, 0.007] |
|  | 7 | 0.84 (0.02) | 0.64 (0.01) | -0.203 | [-0.244, -0.161] | 0.77 (0.02) | 0.69 (0.01) | -0.079 | | [-0.120, -0.037] |
|  | 8 | 0.84 (0.02) | 0.64 (0.01) | -0.197 | [-0.240, -0.154] | 0.76 (0.02) | 0.70 (0.01) | -0.063 | | [-0.106, -0.020] |

**Table S8**

*Results for the RI-CLPM for the main model using the complete case sample (n = 5394)*

|  |  | Unconditional | Condition on sex |
| --- | --- | --- | --- |
| Cross-lagged effects | | | |
| PNeg → Ext | 4yr → 7yr | 0.058 (0.184)  p = 0.752 | 0.032 (0.142)  p = 0.822 |
|  | 7yr → 8yr | 0.195 (0.059)  p = 0.001 | 0.162 (0.050)  p = 0.001 |
|  |  |  |  |
| Ext → PNeg | 4yr → 7yr | 0.062 (0.103)  p = 0.546 | 0.087 (0.102)  p = 0.391 |
|  | 7yr → 8yr | 0.146 (0.047)  p = 0.002 | 0.152 (0.052)  p = 0.003 |
| Autoregressive effects | | | |
| PNeg → PNeg | 4yr → 7yr | 0.622 (0.140)  p < 0.001 | 0.608 (0.129)  p < 0.001 |
|  | 7yr → 8yr | 0.779 (0.056)  p < 0.001 | 0.787 (0.055)  p < 0.001 |
|  |  |  |  |
| Ext → Ext | 4yr → 7yr | 0.544 (0.193)  p = 0.005 | 0.572 (0.172)  p = 0.001 |
|  | 7yr → 8yr | 0.804 (0.066)  p < 0.001 | 0.817 (0.064)  p < 0.001 |

*Note.* Unstandardized coefficients for cross-lagged and autoregressive effects are reported followed by standard errors in parentheses. PNeg = parental negativity; Ext = externalizing problems.

**Table S9**

*Main model results for fully adjusted model*

|  |  | Estimate | S.E. | P-value |
| --- | --- | --- | --- | --- |
| **Measurement models for within-person latent variables** | | | | |
| NEGP_4 by | WILLS_4 | 1.000 | 0.000 | - |
|  | MESS_4 | 0.408 | 0.070 | <0.001 |
|  | NERVES_4 | 0.831 | 0.041 | <0.001 |
|  |  |  |  |  |
| NEGP_7 by | WILLS_7 | 1.000 | 0.000 | - |
|  | MESS_7 | 0.806 | 0.051 | <0.001 |
|  | NERVES_7 | 0.831 | 0.041 | <0.001 |
|  |  |  |  |  |
| NEGP_8 by | WILLS_8 | 1.000 | 0.000 | - |
|  | MESS_8 | 0.806 | 0.051 | <0.001 |
|  | NERVES_8 | 0.831 | 0.041 | <0.001 |
|  |  |  |  |  |
| EXT_4 by | COND_4 | 1.000 | 0.000 | - |
|  | HYP_4 | 1.774 | 0.069 | <0.001 |
|  |  |  |  |  |
| EXT_7 by | COND_7 | 1.000 | 0.000 | - |
|  | HYP_7 | 1.774 | 0.069 | <0.001 |
|  |  |  |  |  |
| EXT_8 by | COND_8 | 1.000 | 0.000 | - |
|  | HYP_8 | 1.774 | 0.069 | <0.001 |
| **Auto-regressive and cross-lagged parameters** | | | | |
|  |  |  |  |  |
| NEGP_8 on | NEGP_7 | 0.783 | 0.053 | <0.001 |
|  | EXT_7 | 0.124 | 0.046 | 0.007 |
|  |  |  |  |  |
| EXT_8 on | NEGP_7 | 0.163 | 0.048 | 0.001 |
|  | EXT_7 | 0.803 | 0.058 | <0.001 |
|  |  |  |  |  |
| NEGP_7 on | NEGP_4 | 0.614 | 0.113 | <0.001 |
|  | EXT_4 | 0.031 | 0.080 | 0.698 |
|  |  |  |  |  |
| EXT_7 on | NEGP_4 | 0.011 | 0.129 | 0.933 |
|  | EXT_4 | 0.558 | 0.155 | <0.001 |
| **Residual covariances between Random Intercepts** | | | | |
| RI_WILLS with | RI_MESS | -0.021 | 0.068 | 0.755 |
|  | RI_NERV | 0.127 | 0.084 | 0.131 |
|  | RI_COND | 0.374 | 0.086 | 0.000 |
|  | RI_HYP | 0.178 | 0.153 | 0.244 |
| RI_MESS with | RI_NERV | 0.216 | 0.059 | 0.000 |
|  | RI_COND | 0.095 | 0.059 | 0.109 |
|  | RI_HYP | 0.083 | 0.105 | 0.427 |
| RI_COND with | RI_NERV | 0.225 | 0.073 | 0.002 |
|  | RI_HYP | 0.435 | 0.190 | 0.022 |
| RI_HYP with | RI_NERV | 0.215 | 0.129 | 0.097 |
| **Residual variances for Random Intercepts** | | | | |
|  | RI_COND | 0.643 | 0.109 | 0.000 |
|  | RI_HYP | 1.993 | 0.343 | 0.000 |
|  | RI_WILLS | 0.276 | 0.101 | 0.006 |
|  | RI_MESS | 0.349 | 0.051 | 0.000 |
|  | RI_NERV | 0.415 | 0.074 | 0.000 |
| **Covariances (wave 4) and residual covariances (waves 7 and 8) for cross-sectional within-person latent variables** | | | | |
| NEGP_4 with | EXT_4 | 0.402 | 0.087 | 0.000 |
| NEGP_7 with | EXT_7 | 0.214 | 0.017 | 0.000 |
| NEGP_8 with | EXT_8 | 0.135 | 0.011 | 0.000 |
| **Variances (wave 4) and residual variances (waves 7 and 8) for within-person latent variables** | | | | |
|  | NEGP_4 | 0.513 | 0.102 | 0.000 |
|  | EXT_4 | 0.530 | 0.110 | 0.000 |
|  | NEGP_7 | 0.338 | 0.023 | 0.000 |
|  | NEGP_8 | 0.161 | 0.019 | 0.000 |
|  | EXT_7 | 0.392 | 0.028 | 0.000 |
|  | EXT_8 | 0.154 | 0.015 | 0.000 |

*Note.* Parameters not shown (in the interest of conciseness) – measurement models for random intercepts (all loadings fixed to 1.0), residual variances for manifest items representing externalising problems, thresholds for manifest binary indicators of negative parenting, item intercepts for manifest items representing externalising problems, parameters representing regression of wave 7 and 8 within-person latent variables on child sex, and parameters representing regression of each random intercept on each of the binary indices of family adversity.

**References**

Asparouhov, T. & Muthén, B. (2006). Robust Chi Square Difference Testing with Mean and Variance Adjusted Test Statistics. *Mplus Web Notes*: *No. 10.* <https://www.statmodel.com/download/webnotes/webnote10.pdf>

Bongers, I. L., Koot, H. M., Van Der Ende, J., & Verhulst, F. C. (2004). Developmental trajectories of externalizing behaviors in childhood and adolescence. *Childddevelopment*, *75*(5), 1523-1537. <https://doi.org/10.1111/j.1467-8624.2004.00755.x>

Curran, P. J., & Bauer, D. J. (2011). The disaggregation of within-person and between-person effects in longitudinal models of change. *Annual Review of Psychology*, *62*, 583-619. <https://doi.org/10.1146/annurev.psych.093008.100356>

Hamaker, E. L., Kuiper, R. M., & Grasman, R. P. P. P. (2015). A critique of the cross-lagged panel model. *Psychological Methods*, *20*(1), 102–116. <https://doi.org/10.1037/a0038889>

Hoffman, L. Testing Measurement Invariance across Groups in Item Factor Models in Mplus version 7.11. Retrieved 1st May 2024, <https://www.lesahoffman.com/PSYC948/948_Example9c_IFA_Multiple_Group_Invariance.pdf>

Marçal, K.E. Demographic and Socioeconomic Predictors of Behavioral Trajectories from Age 3 to 15: A Longitudinal Mixed Effects Approach. *J Child Fam Stud* **29**, 1818–1832 (2020). <https://doi.org/10.1007/s10826-020-01710-8>

Mulder, J. D., & Hamaker, E. L. (2021). Three Extensions of the Random Intercept Cross-Lagged Panel Model. *Structural Equation Modeling: A Multidisciplinary Journal*, *28*(4), 638-648. <https://doi.org/10.1080/10705511.2020.1784738>

Mund, M., Johnson, M. D., & Nestler, S. (2021). Changes in Size and Interpretation of Parameter Estimates in Within-Person Models in the Presence of Time-Invariant and Time-Varying Covariates. *Frontiers in psychology*, *12*, 666928. <https://doi.org/10.3389/fpsyg.2021.666928>
